# Editorial: The impact of endometriosis

**DOI:** 10.3389/fgwh.2023.1190974

**Published:** 2023-05-22

**Authors:** Adriana Luckow Invitti, Lysia Demetriou

**Affiliations:** ^1^ Setor de Algia Pélvica e Endometriose, Departamento de Ginecologia, Escola Paulista de Medicina, Universidade Federal de São Paulo, São Paulo, Brazil; ^2^ Oxford Endometriosis CaRe Centre, Nuffield Department of Women’s and Reproductive Health, Medical Science Division, University of Oxford, Oxford, United Kingdom; ^3^ Pain in Women, Nuffield Department of Women’s and Reproductive Health, Medical Science Division, University of Oxford, Oxford, United Kingdom

**Keywords:** endometriosis, awareness, women’s health, quality of life, chronic illness, delay diagnosis, treatment

**Editorial on the Research Topic** The impact of endometriosis

Worldwide, endometriosis affects more than 190 million women and those assigned female at birth. This corresponds to 10% of women in reproductive age (1) who often experience clinical symptoms beyond menopause (2). Individuals suffering from the disorder may experience devastating effects on their quality of life, impacting both their physical and mental health resulting in the inability to perform even simple everyday tasks (3). Endometriosis also impacts people in the sufferer's environment including partners, family and work colleagues (2). One of the most common symptoms is disabling chronic pelvic pain, however, endometriosis is also the leading cause of infertility amongst women (1, 4, 5). Although, it is difficult to calculate endometriosis' direct economic impact, it is comparable to type 2 diabetes, rheumatoid arthritis and Crohn's disease (6, 7). The aim of this Research Topic was to present research that provides insights in the impact of the disease as well as promote endometriosis awareness.

Following this objective, Ellis et al. provide an updated summary on endometriosis research and available diagnostic tools and treatments in clinics. They discuss the currently available funding in the field that falls short behind other chronic pain conditions such as Crohn's disease, leading to delayed advancements in the endometriosis field and increased economic burden for the patients. The authors strongly advocated that increased funding in endometriosis would benefit the advancements in diagnosis and treatment particularly regarding currently evolving research fields such as new biomarker analysis, nanomedicine and microbiota composition analysis. There is much space for improvement regarding endometriosis awareness as well as the availability of multidisciplinary treatments and diagnostic delay and accuracy. Many public health policies have been recently published to improve the access to treatment. Public awareness and professional training for an earlier diagnosis of the disease have also been increased. However, there is still a long diagnostic delay of approximately 8–12 years (2). Additionally, the disability-adjusted life-years (DALYs) of endometriosis has increased between 1990 and 2019 (8). The scientific community have been working to standardize the research on endometriosis (9–12]) and increase collaborations with public engagement charities and organizations to improve awareness. In 2017, a consortium of endometriosis researchers published the global priorities for endometriosis research (13). These initiatives are being translated into an increasing number of collaborative works across the world, including mid and low income countries. These, help in improving the quality of the results to better reflect disease management and patients' quality of life (14).

The collection of articles in this Research Topic includes the recent *Danish translation and cross-cultural adaptation of the World Endometriosis Research Foundation (WERF) Endometriosis Phenome and Biobanking Harmonization project (EPHect) Endometriosis Patient Questionnaire*. This questionnaire has already been translated into 15 languages (Arabic, Assamese, Chinese, Dutch/Flemish, French, German, Hindi, Hispanic, Italian, Kannada, Malayalam, Marathi, Portuguese, Spanish, and Turkish)^1^. The translation and cultural adaptations of this questionnaire permits the comparison between different populations without the bias of data collection. These initiatives on standardization leads to more robust results that can include different populations and improve the understanding of the disease and its management that can differ between different populations.

Following this, the article on *The experiences of endometriosis patients with diagnosis and treatment in New Zealand* highlights the views of patients on endometriosis management. The patient's perspective on diagnosis and their treatments is crucial in adjusting the management to the needs of this population. It emphasizes some well-known problems in endometriosis management such as the diagnostic delay as well as the inefficacy of pain killers and hormonal treatments offered as a first and sometimes only treatment for the pain management. This article brings attention to the fact that the current standard treatments are not effective to every patient and highlights the urgent need for the development of new treatments including an interdisciplinary approach to manage endometriosis symptoms (15).

Considering this, Nieves-Vázquez et al., present possibly the first feasibility pilot study on an Environmental Enrichment (EE) intervention for people with endometriosis. EEs involve the establishment of changes in patients' environment which encourage exercise and socialization combined with sensory and cognitive stimulations and primary impairment therapy (16). They are particularly important interventions regarding quality of life and impact of chronic disorders on the patients' wellbeing. However, to-date only pre-clinical EE intervention studies exist for endometriosis. Even though the study was conducted mid-COVID-19 pandemic lockdowns and earthquakes in Puerto Rico, it does provide encouraging results regarding the feasibility of EE interventions with higher than 40% enrolment and adherence rates. The results suggest that such studies are feasible and much needed as more than 95% of the participants in the intervention group reported that they would recommend the intervention to other people with endometriosis and more than 80% of them reported that they would regularly participate in such interventions. Furthermore, this study exposed both the challenges and opportunities for improvement for future RCT trials employing EE.

Another important gap on endometriosis treatment regards the availability of different drugs to treat the disease. Chung and Han reviewed angiogenesis in the pathophysiology of endometriosis and discuss the pros and cons of the anti-angiogenic treatments in substitution to the current hormonal treatments. The authors summarized all the anti-angiogenic treatments currently being tested in pre-clinical and clinical trials (e.g., dopamine agonists, COX-2 inhibitors, growth factor inhibitors, statins, among others). This review points out the putative benefits of anti-angiogenic treatments, as well as the main expected side effects and their impacts. Like other articles in this collection, this paper highlights the need for a multidisciplinary approach to endometriosis management.

This Research Topic has received contributions regarding endometriosis awareness and highlighted the existing gaps in the understanding of the disease, its management, as well as the perception of endometriosis patients' on their treatments. It has been made clear that there is a need for more multidisciplinary research into the mechanisms underlying endometriosis as well as the development of new treatments and interventions. We believe that the patient perspective should be prioritized when designing new treatment studies. However, given that this is an underfunded research area, more awareness is required to direct more funding and resources into the field by governments and funding bodies.

## Footnotes

^1^www.endometriosisfoundation.org – Accessed March 6, 2023.
